# TGR5 deficiency aggravates hepatic ischemic/reperfusion injury via inhibiting SIRT3/FOXO3/HIF-1ɑ pathway

**DOI:** 10.1038/s41420-020-00347-2

**Published:** 2020-11-01

**Authors:** Qi Wang, Song Wei, Lei Li, Jiannan Qiu, Shun Zhou, Chengyu Shi, Yong Shi, Haoming Zhou, Ling Lu

**Affiliations:** 1 Jiangsu Cancer Hospital, Jiangsu Institute of Cancer Research, Nanjing Medical University Affiliated Cancer Hospital, The First Affiliated Hospital of Nanjing Medical University, Research Unit of Liver Transplantation and Transplant Immunology, Chinese Academy of Medical Sciences, NHC Key Laboratory of Living Donor Liver Transplantation, Nanjing, China; 2 Key Laboratory of Liver Transplantation, Chinese Academy of Medical Sciences, NHC Key Laboratory of Living Donor Liver Transplantation, Nanjing, China; 3 School of Medicine, Southeast University, Nanjing, China; 4 Jiangsu Key Lab of Cancer Biomarkers, Prevention and Treatment, Collaborative Innovation Center for Personalized Cancer Medicine, Nanjing Medical University, Nanjing, China; 5 State Key Laboratory of Reproductive Medicine, Nanjing, China

**Keywords:** Inflammation, Transplant immunology

## Abstract

Ischemia/reperfusion (I/R) injury is responsible for liver injury during hepatic resection and liver transplantation. The plasma membrane-bound G protein-coupled bile acid receptor (TGR5) could regulate immune response in multiple liver diseases. Nevertheless, the underlying role of TGR5 in hepatic I/R injury remains largely unknown. This study aimed to investigate the potential mechanism of TGR5 in hepatic I/R injury. Wild-type (WT) and TGR5 knockout (TGR5KO) mice were used to perform hepatic I/R, and macrophages were isolated from mice for in vitro experiments. The results demonstrated that knockout of TGR5 in mice significantly exacerbated liver injury and inflammatory response. TGR5KO mice infused with WT macrophages showed relieved liver injury. Further study revealed that TGR5 knockout inhibited sirtuin 3 (SIRT3) and forkhead box O3 (FOXO3) expression. In vitro experiments indicated that SIRT3 inhibited acetylation, ubiquitination and degradation of FOXO3. FOXO3 inhibited HIF-1α transcription by binding to its promoter. TGR5 knockout inhibited SIRT3 expression, thus promoted the acetylation, ubiquitination, and degradation of FOXO3, which resulted in increased HIF-1α transcription and macrophages proinflammatory response. Collectively, TGR5 plays a critical protective role in hepatic I/R injury. FOXO3 deacetylation mediated by SIRT3 can attenuate hepatic I/R injury. TGR5 deficiency aggravates hepatic I/R injury via inhibiting SIRT3/FOXO3/HIF-1α pathway.

## Introduction

Ischemia/reperfusion injury is a local inflammation response dominated by innate immunity. It is an important cause of liver dysfunction in liver transplantation^1^. Exogenous danger signals such as danger-associated molecular patterns (DAMPs) or pathogen-derived molecular patterns (PAMPs) released from injured cells is recognized by innate immune cells^2–4^. Macrophages, the important innate immune cells, are sensitive to DAMPs and can result in liver inflammatory response^5^.

The plasma membrane-bound G protein-coupled bile acid receptor (TGR5) expression varied differently in multiple tissues^6–8^. Increasing evidences had demonstrated that TGR5 could modulate bile acid homeostasis, energy expenditure, and glucose metabolism^9–11^. A study indicated that TGR5 activation inhibited kidney disease in obesity and diabetes by inhibiting oxidative stress via promoting sirtuin 1 (SIRT1) and sirtuin 3 (SIRT3) expression^12^. Furthermore, TGR5 plays a vital role in anti-inflammatory response. A previous study showed that TGR5 activation in vivo decreased lipopolysaccharide (LPS)–induced inflammatory response in liver^13^. In vitro studies indicated that TGR5 might suppress the function of macrophages treated with bile acid^14,15^. Another study demonstrated that TGR5 might alleviate murine hepatic chronic inflammation via inhibiting nuclear factor kappa B (NF-kB)^16^. However, the underlying role of TGR5 in hepatic I/R injury and the mechanism TGR5 regulating immune and inflammatory response remains largely unknown.

SIRT3 is a highly conserved nicotinamide adenine dinucleotide (NAD+)-dependent deacetylase that mainly expressed in mitochondria^17–19^. Studies showed that SIRT3 deacetylated most of the mitochondrial proteins, such as the forkhead box O transcription factor 3 (FOXO3), manganese superoxide dismutase (MnSOD), and cyclophilin D (CypD)^20–24^. Another study also revealed that SIRT3 might modulate HIF-1α stabilization^25^. SIRT3 also indirectly inhibits HIF-1α transcriptional activity and the activation of HIF-1α by reducing reactive oxygen species (ROS) production^26,27^. Thus, SIRT3 can regulate various cellular biological conditions, such as mitochondrial dynamics, and oxidative stress via altering proteins acetylation. Studies demonstrated that SIRT3 alleviated tissue injury caused by cerebral^28^, myocardial^29^, and limb^30^ I/R. Study had indicated that TGR5 played a protective role in nephropathy in obesity and diabetes via promoting SIRT3 expression^12^. The role of SIRT3 in hepatic I/R injury remains poorly known. Therefore, we hypothesized that TGR5 attenuated the hepatic I/R injury by promoting SIRT3 expression thus inhibiting the acetylation and ubiquitination of FOXO3.

Hypoxia inducible factor-1 (HIF-1) is a transcription factor consist of an inducibly expressed HIF-1α subunit and a constitutively expressed HIF-1β subunit. Under normoxic conditions, HIF-1α is constantly synthesized and degraded by HIF prolyl hydroxylase (PHD). Under hypoxic conditions, HIF-1α degradation decreased, thus facilitating the transcription of numerous genes involved in cellular responses to oxygen deprivation^31^. HIF-1α plays a vital role in regulating innate immune cells. Myeloid-specific HIF-1α disruption inhibited inflammation via suppressing macrophage aggregation and invasion^32^. A previous study revealed that HIF-1 activation by pretreatment with PHD inhibitor exacerbated murine renal I/R injury^33^. Another study showed that FOXO3 activation abolished HIF-1α induction by hypoxia^34^. FOXO3 is stress responsive and prevents HIF-1α stabilization and ROS accumulation to reduce HIF-1α-induced apoptosis during hypoxia^35^. However, the specific roles of FOXO3 and HIF-1α in hepatic I/R injury remain largely unclear. Thus, we speculated that FOXO3 regulated hepatic I/R injury by suppressing HIF-1α expression. TGR5 deficiency aggravated hepatic ischemic/reperfusion injury via inhibiting SIRT3/FOXO3/HIF-1α axis.

The objectives of this study were to determine the vital role of TGR5 in hepatic I/R injury and explore the potential mechanism of TGR5 regulating hepatic I/R injury.

## Results

### **TGR5 deficiency exacerbates hepatic I/R** injury

**Fig. 1:**
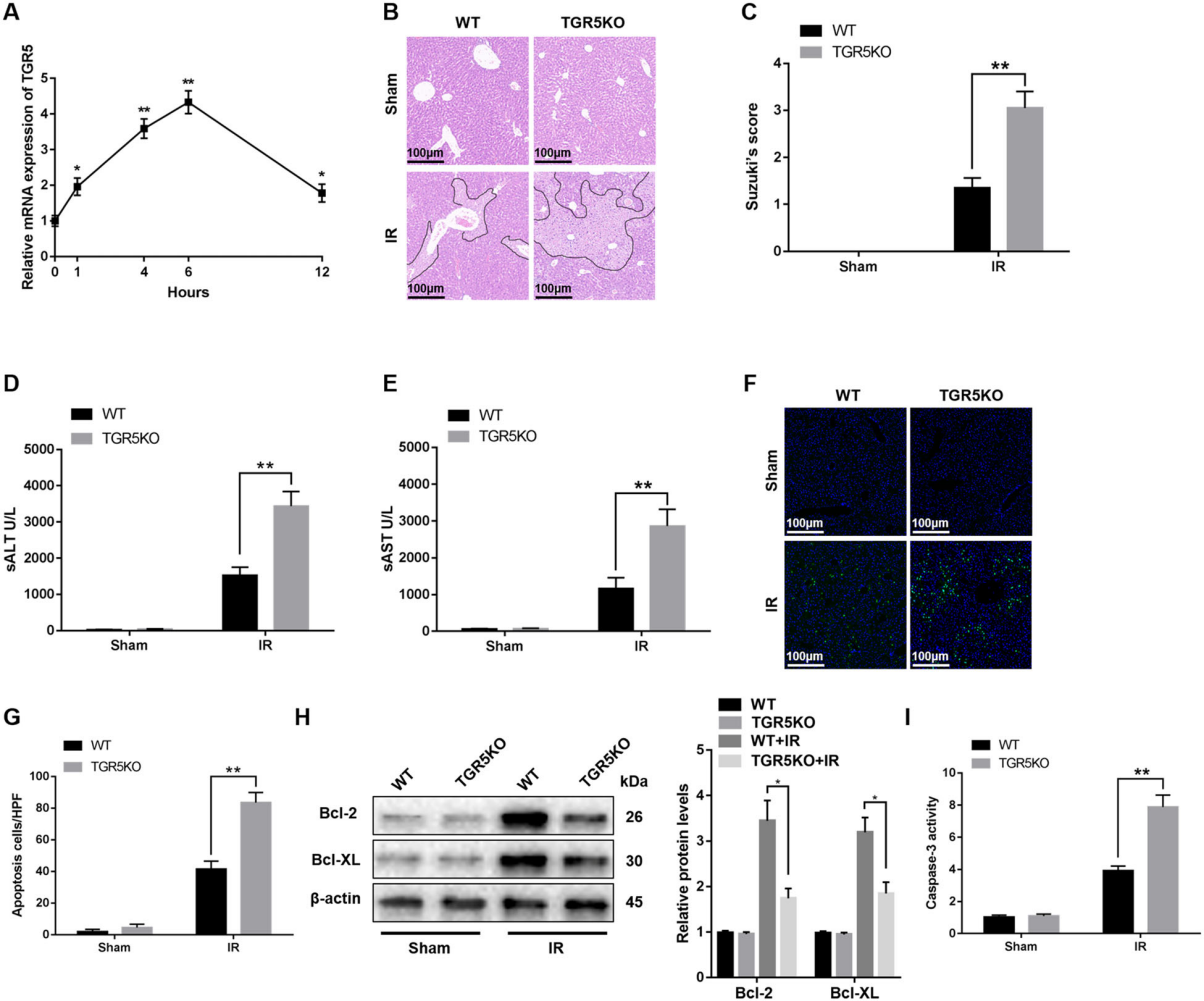
TGR5 deficiency exacerbates hepatic ischemia/reperfusion injury. **a** The expression of TGR5 in liver tissues from WT mice after ischemia and different periods of reperfusion (1, 4, 6, and 12 h) were determined by qRT-PCR, value were normalized against sham group, *n* = 6 mice at each time point. WT and TGR5KO mice were performed with 90 min of partial liver ischemia and 6 h of reperfusion, respectively. **b** Livers harvested after 6 h reperfusion were subjected to histopathology. Representative of six mice/group. **c** The damage of liver architecture was evaluated by Suzuki’s score. *n* = 6 mice/group. **d**, **e** Hepatocellular function was detected by serum ALT (U/L) and AST (U/L). *n* = 6 mice/group. **f** Liver sections were performed with TUNEL staining (original magnification ×200). Nuclear was stained with DAPI. Representative of six mice/group. **g** TUNEL-positive cells were quantified in different experimental groups (original magnification ×200). *n* = 6 mice/group. **h** Western blots were performed to analyze Bcl-2, Bcl-XL, and β-actin protein levels in liver tissues. Representative of three experiments. **i** Caspase-3 activity assay was performed to determine cellular activity. *n* = 6 mice/group. The values were showed as the mean ± SEM; **P < 0.01; *P < 0.05.

WT mice were performed with a sham operation or 90 min of ischemia with various reperfusion times (1, 4, 6, and 12 h). The mRNA levels of TGR5 in the liver tissues were detected. Figure 1a showed that the transcription levels of TGR5 increased in the early phase of reperfusion and peaked at 6 h after reperfusion. Then, liver tissues from mice performed with 90 min of warm ischemia followed by 6 h of reperfusion were used to assess hepatocellular function. The livers from TGR5KO mice showed severer damage compared to the livers of WT mice (Fig. 1b, c). The levels of serum alanine aminotransferase (ALT) and aspartate transaminase (AST) in TGR5KO mice were significantly higher compared to the WT group (Fig. 1d, e). Consistent with these data, TUNEL positive cells in ischemic livers were higher in TGR5KO mice compared to the WT group (Fig. 1f, g). The levels of anti-apoptotic proteins, Bcl-2 and Bcl-XL, were decreased in TGR5KO ischemic livers compared to the WT group (Fig. 1h). Increased caspase-3 activity in TGR5KO group but not in WT group further confirmed above results (Fig. 1i). These results indicated that TGR5 knockout exacerbated IR-induced liver damage.

### TGR5 deficiency increases macrophage/neutrophil trafficking

**Fig. 2:**
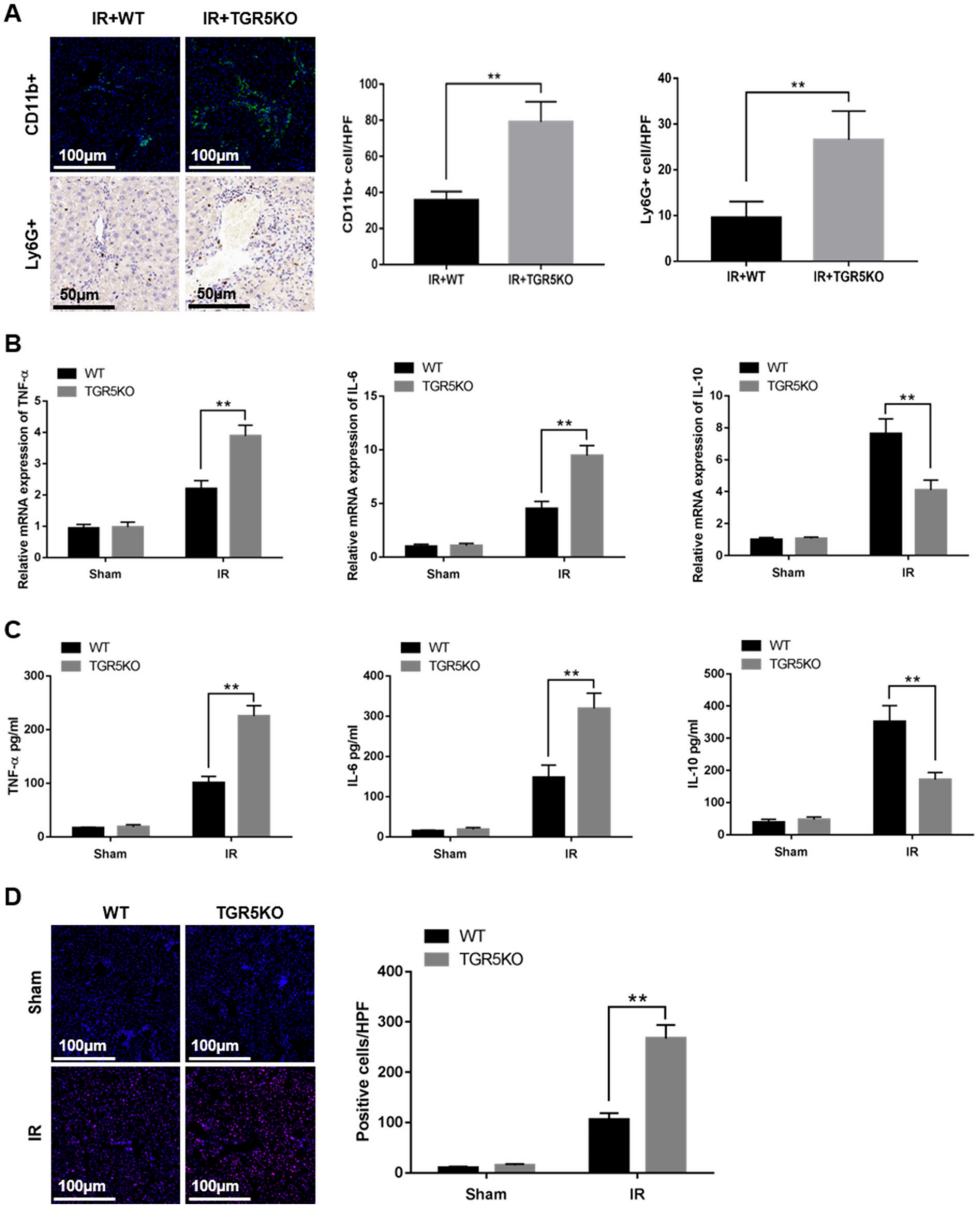
TGR5 deficiency increases macrophage/neutrophil trafficking. **a** CD11b+ macrophages in liver were detected by immunofluorescence and Ly6G+ neutrophils in liver were determined by immunohistochemistry. Results were quantified in different groups (original magnification ×200 or ×400). Representative of six mice/group. **b** Quantitative RT-PCR was performed to detect inflammatory gene in liver tissues, *n* = 6 mice/group. **c** Enzyme-linked immunosorbent assay (ELISA) was performed to detect serum levels of inflammatory cytokines, *n* = 6 mice/group. **d** The levels of ROS in liver tissues was examined by DHE (original magnification ×200). Representative of six mice/group. Cells labeled with red fluorescent were counted blindly in 10 HPF/section (original magnification ×200). The numbers of ROS-producing cells (red) per high power field were quantified (original magnification ×200), *n* = 6 mice/group. Mean ± SEM; **P < 0.01; *P < 0.05.

To explore whether TGR5 affects the recruitment of inflammatory cell in ischemic livers, immunofluorescence was used to detect CD11b+ macrophages and immunohistochemistry was used to detect Ly6G+ neutrophils. Ischemic livers from TGR5KO mice showed increased CD11b+ macrophages and Ly6G+ neutrophils compared to the WT group (Fig. 2a). TGR5KO upregulated proinflammatory TNF-α and IL-6 and downregulated anti-inflammatory IL-10 expression in ischemic livers compared to the WT group (Fig. 2b, c). TGR5KO increased ROS levels in ischemic livers compared to the WT group (Fig. 2d). These results demonstrated that TGR5 knockout increased macrophage/neutrophil infiltration.

### TGR5 overexpression ameliorates hepatic I/R injury

**Fig. 3:**
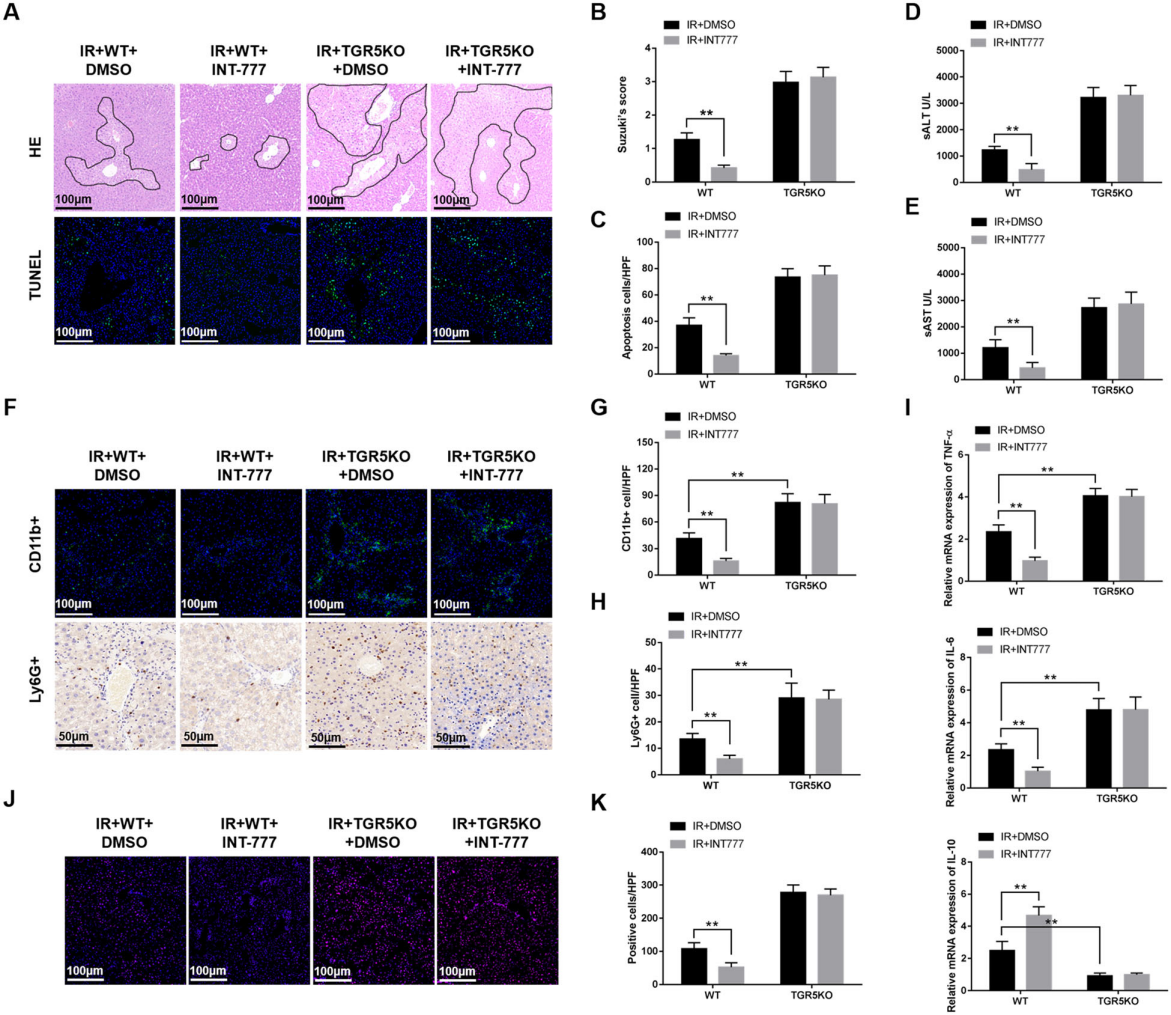
TGR5 overexpression ameliorates hepatic ischemia/reperfusion injury. **a** Livers were harvested after 6 h reperfusion and were subjected to histopathologic analysis. liver sections were performed with TUNEL staining (original magnification ×200). Nuclear was stained with DAPI. Representative of six mice/group. **b** The damage of liver architecture was analyzed by Suzuki’s score. *n* = 6 mice/group. **c** TUNEL-positive cells were quantified in different experimental groups (original magnification ×200), *n* = 6 mice/group. **d**, **e** Serum ALT (U/L) and AST (U/L) were performed to examine hepatocellular function. *n* = 6 mice/group. **f** Immunofluorescence was performed to detect CD11b+ macrophages in liver and immunohistochemistry was performed to detect Ly6G+ neutrophils in liver. Representative of six mice/group. **g** CD11+ bcells were quantified in different groups (original magnification ×200), *n* = 6 mice/group. **h** Ly6G+ cells were quantified in different groups (original magnification ×200), *n* = 6 mice/group. **i** Quantitative RT-PCR was performed to detect inflammatory gene in liver tissues, *n* = 6 mice/group. **j** The levels of ROS in liver tissues was examined by DHE (original magnification ×200). Representative of six mice/group. **k** The numbers of ROS-producing cells (red) per high power field were quantified (original magnification ×200), *n* = 6 mice/group. Mean ± SEM; **P < 0.01; *P < 0.05.

To further confirm the role of TGR5 in IR-induced liver injury, TGR5 agonist INT-777 was used to overexpress TGR5. IR+WT+INT-777 group showed decreased liver architecture damage and TUNEL positive cells in ischemic livers compared to the IR+TGR5KO+INT-777 group (Fig. 3a–c). The levels of serum ALT and AST were significantly increased in IR+TGR5KO+INT-777 group compared to the IR+WT+INT-777 group (Fig. 3d, e). Immunofluorescence and immunohistochemistry further indicated that CD11b+ macrophages and Ly6G+ neutrophils were decreased in IR+WT+INT-777 group but not in IR+TGR5KO+INT-777 group (Fig. 3f–h). IR+WT+INT-777 group showed decreased proinflammatory TNF-α and IL-6 and increased anti-inflammatory IL-10 expression compared to the IR+TGR5KO+INT-777 group (Fig. 3i). The level of ROS in IR+WT+INT-777 group was significantly decreased compared to the IR+TGR5KO+INT-777 group (Fig. 3j, k). Taken together, these results suggested that TGR5 overexpression ameliorated hepatic IR injury.

### TGR5 deficiency promotes macrophage inflammatory response and oxidative stress via suppressing SIRT3 expression

**Fig. 4:**
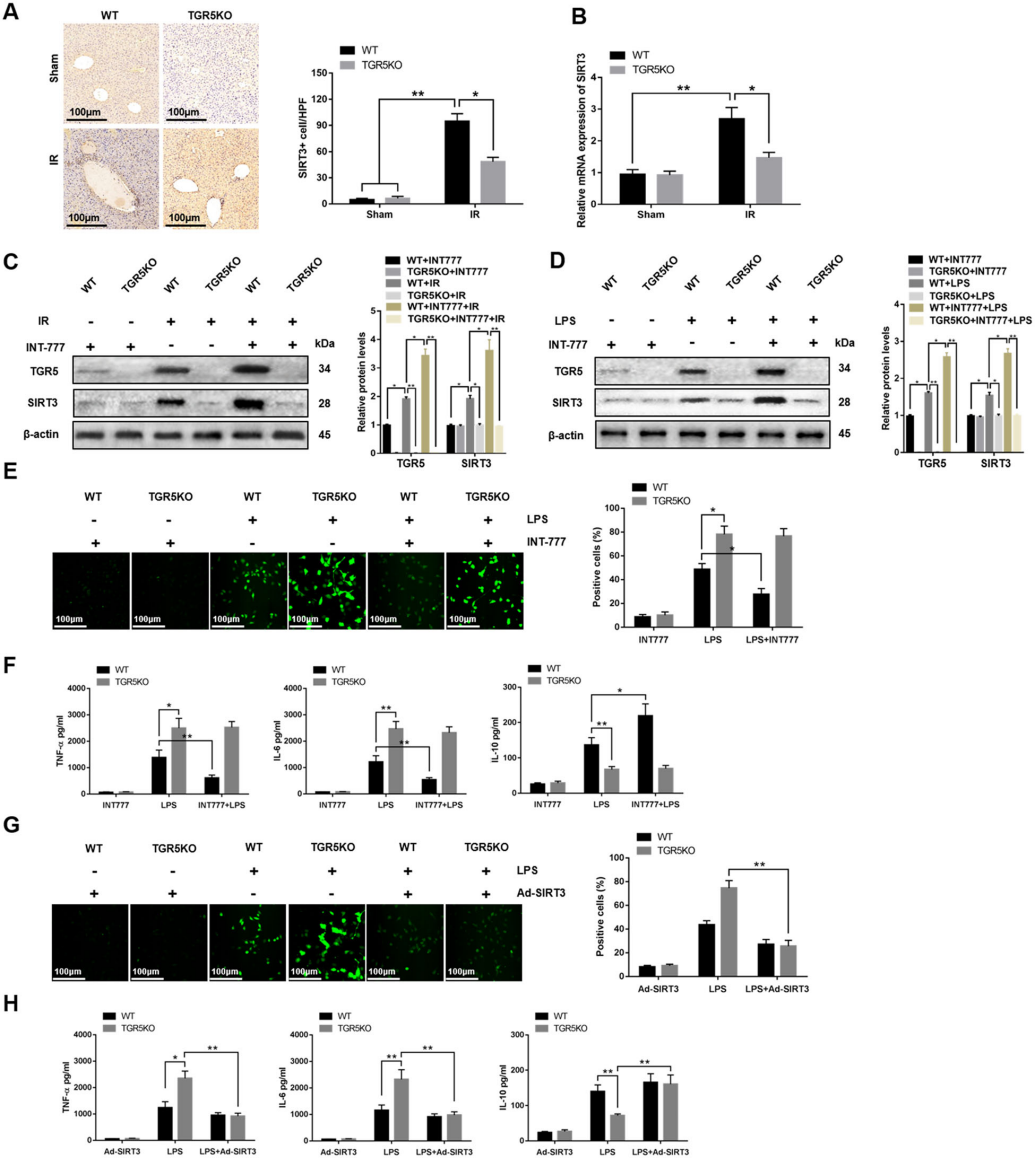
TGR5 deficiency promotes macrophage inflammatory response and oxidative stress via suppressing SIRT3 expression. **a** Immunohistochemistry was performed to detect SIRT3 in murine liver tissues (original magnification ×200) Representative of six mice/group. SIRT3+ cells were quantified in different groups (original magnification ×200), *n* = 6 mice/group. **b** SIRT3 gene expression in liver tissues was measured by quantitative RT-PCR. *n* = 6 mice/group. **c** Western blots were carried out to analyze the levels of TGR5 and SIRT3 in liver tissues. Representative of three experiments. **d** Western blots were carried out to analyze the levels of TGR5 and SIRT3 in BMDMs. Representative of three experiments. **e** The levels of ROS in BMDMs was examined by DCFH-DA (original magnification ×200). Representative of three experiments. Quantification of ROS-producing cells (green) per high power field (original magnification ×200). *n* = 3/group. **f** Inflammatory gene expression in supernatant was measured by ELISA. *n* = 3/group. **g** ROS production was detected by DCFH-DA in BMDMs (original magnification ×200). Representative of three experiments. The numbers of ROS-producing cells (green) per high power field were quantified (original magnification ×200), *n* = 3/group. **h** Inflammatory gene expression in supernatant was detected by ELISA. *n* = 3/group. Mean ± SEM; **P < 0.01; *P < 0.05.

The mechanisms how TGR5 deficiency aggravates IR-induced liver injury remains largely unknown. A previous study indicated that TGR5 agonist INT-777 increased SIRT3 expression, a mitochondrial protein deacetylase, and prevented renal oxidative stress^12^. Whether TGR5 influences SIRT3 expression in hepatic IR injury remains unknown. Immunohistochemistry demonstrated that the expression of SIRT3 in IR+TGR5KO group was decreased compared to the IR+WT group, as evidenced by qRT-PCR (Fig. 4a, b). Western blot further showed that INT-777 increased SIRT3 expression in liver tissues from IR+WT group compared to the IR+TGR5KO group (Fig. 4c). In vitro, WT and TGR5KO BMDMs were treated with LPS and INT-777. The expression of SIRT3 was significantly increased in LPS+WT+INT-777 group compared to the LPS+TGR5KO+INT-777 group (Fig. 4d). Figure 4e showed that ROS levels in BMDMs was decreased in LPS+WT+INT-777 group compared to the LPS+TGR5KO+INT-777 group. Proinflammatory TNF-α and IL-6 expression was downregulated and anti-inflammatory IL-10 expression was upregulated in LPS+WT+INT-777 group compared to the LPS+TGR5KO+INT-777 group (Fig. 4f). SIRT3 overexpression decreased ROS levels and proinflammatory TNF-α and IL-6 expression and increased anti-inflammatory IL-10 expression in LPS+TGR5KO group (Fig. 4g, h). These results suggested that TGR5 deficiency promoted macrophage inflammatory response and oxidative stress via inhibiting SIRT3 expression.

### SIRT3 regulates TGR5-mediated immune response via interacting with FOXO3

**Fig. 5:**
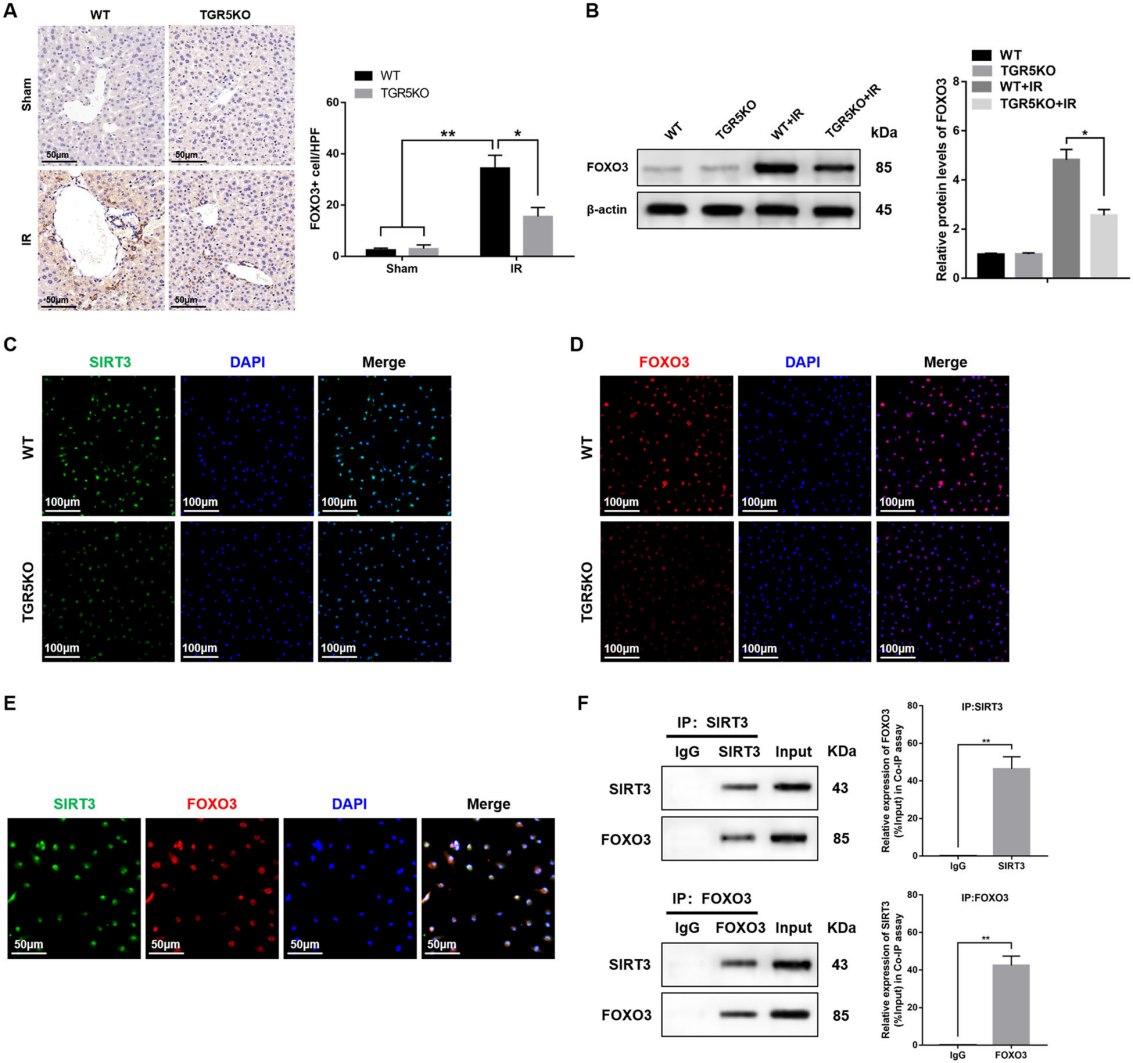
SIRT3 regulates TGR5-mediated immune response via interacting with FOXO3. **a** Immunohistochemistry was performed to detect FOXO3 in murine liver tissues (original magnification ×400), Representative of six mice/group. FOXO3+ cells were quantified in different groups (original magnification ×200), *n* = 6 mice/group. **b** Western blots were carried out to detect the levels of FOXO3 in liver tissues. Representative of three experiments. **c**, **d** Immunofluorescence staining of SIRT3 (green) and FOXO3 (red) in LPS-stimulated BMDMs. Representative of three experiments. **e** Immunofluorescence staining for SIRT3 (green) and FOXO3 (red) colocalization were detected in WT BMDMs stimulated with LPS. Representative of three experiments. **f** Immunoprecipitation analysis of SIRT3 and FOXO3 in WT BMDMs stimulated with LPS. Representative of three experiments. Mean ± SEM; **P < 0.01; *P < 0.05.

Previous studies indicated that SIRT3-mediated deacetylation of FOXO3 decreased cellular ROS levels and ameliorated cardiac hypertrophy in mice^37,38^. The association between SIRT3 and FOXO3 in hepatic IR injury remains largely unclear. Immunohistochemistry demonstrated that the expression of FOXO3 in IR+TGR5KO group was decreased compared to the IR+WT group, as evidenced by western blot (Fig. 5a, b). Having demonstrated the importance of both the SIRT3 and FOXO3 in the modulation of hepatic IR injury, we next explored whether there is crosstalk between the SIRT3 and FOXO3 in TGR5-mediated immune regulation. Immunofluorescent staining revealed increased nuclear SIRT3 (Fig. 5c) and FOXO3 (Fig. 5d) expression in WT BMDMs treated with LPS compared to TGR5KO group. Interestingly, both SIRT3 and FOXO3 were colocalized in the WT BMDMs nucleus (Fig. 5e). Moreover, coimmunoprecipitation assays revealed that SIRT3 can bind to endogenous FOXO3 in WT BMDMs (Fig. 5f). Collectively, these results indicated that SIRT3 regulated TGR5-mediated immune response via interacting with FOXO3.

### SIRT3 regulates macrophage oxidative stress and inflammatory response by inhibiting the acetylation and ubiquitination of FOXO3

**Fig. 6:**
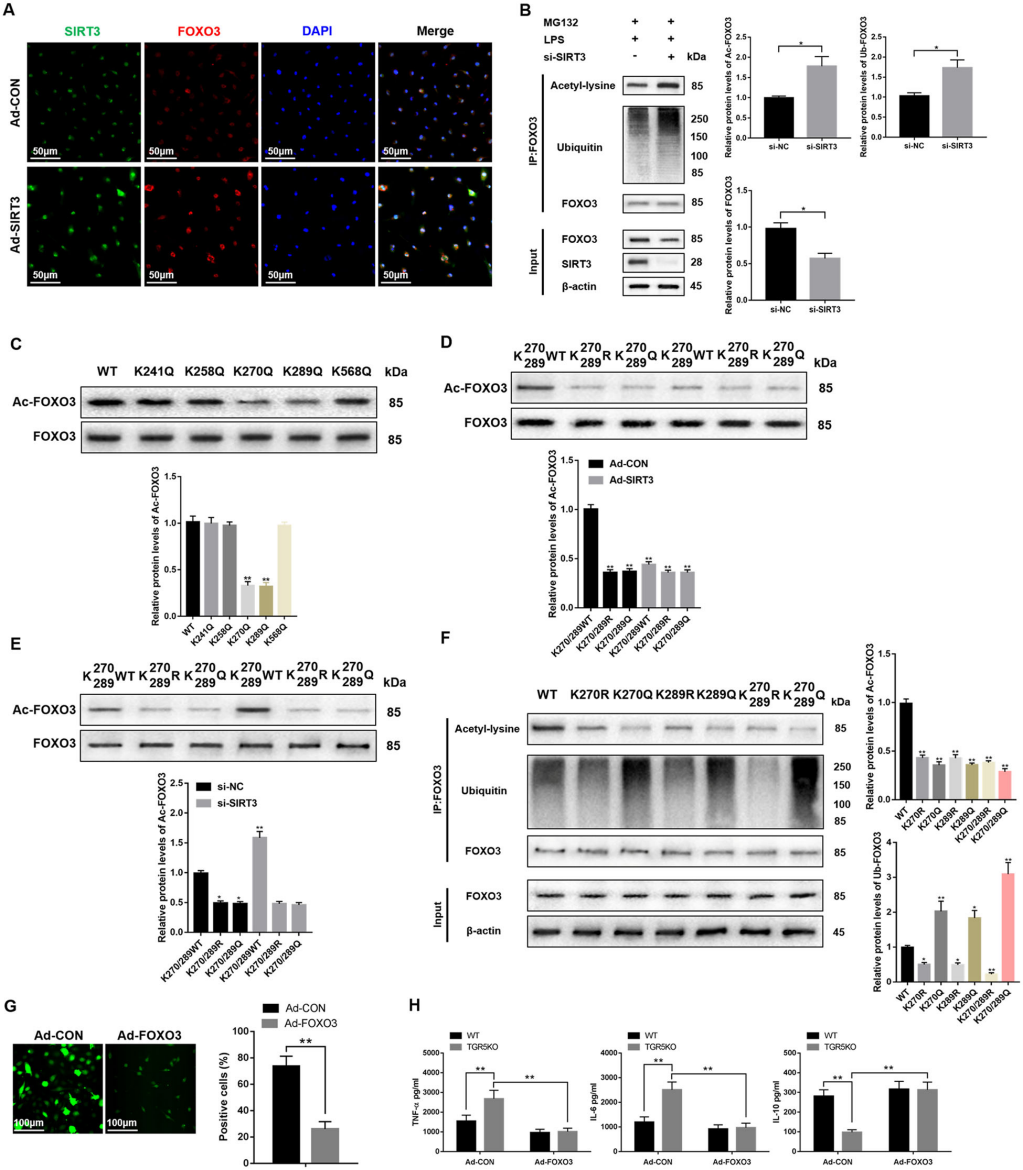
SIRT3 regulates macrophage oxidative stress and inflammatory response by inhibiting the acetylation and ubiquitination of FOXO3. **a** Immunofluorescence staining for SIRT3 (green) and FOXO3 (red) colocalization in LPS-stimulated TGR5KO BMDMs treated with Ad-SIRT3 or Ad-CON. Representative of three experiments. **b** BMDMs were transfected with SIRT3 siRNA for 48 h and stimulated with LPS for another 6 h. Then, MG132 (25 μM) was added for 4 h. The lysates were used for immunoprecipitation with FOXO3, and immunoblot was performed with anti-Acetyl-lysine, anti-Ubiquitin, anti-FOXO3, and anti-SIRT3 antibodies. Representative of three experiments. **c** FOXO3 acetylation was examined in BMDMs transfected with potential acetylation site-specific mutant plasmids of FOXO3. Representative of three experiments. **d** FOXO3 acetylation was detected in BMDMs transfected with Ad-SIRT3 adenoviruses and potential acetylation site-specific mutant plasmids of FOXO3. Representative of three experiments. **e** FOXO3 acetylation was detected in BMDMs transfected with SIRT3 siRNA and potential acetylation site-specific mutant plasmids of FOXO3. Representative of three experiments. **f** Acetylation, ubiquitination and degradation of FOXO3 was examined in BMDMs transfected with FOXO3WT, FOXO3K270R, FOXO3K270Q, FOXO3K289R, FOXO3K289Q, FOXO3K270/289R, or FOXO3K270/289Q mutant plasmids. Representative of three experiments. **g** ROS levels in TGR5KO BMDMs transfected with Ad-SIRT3 adenoviruses (original magnification ×200). Representative of three experiments. The numbers of ROS-producing cells (green) per high power field were quantified (original magnification ×200), *n* = 3/group. **h** The levels of inflammatory gene in supernatant was detected by ELISA. *n* = 3/group. Mean ± SEM; **P < 0.01; *P < 0.05.

To further determine the mechanism how SIRT3 interacts with FOXO3 and regulates TGR5-mediated immune response, we overexpressed SIRT3 in TGR5KO BMDMs before stimulated with LPS. Immunofluorescence colocalization indicated that SIRT3 overexpression promoted FOXO3 expression (Fig. 6a). Figure 6b indicated that SIRT3 knockdown in TGR5KO BMDMs before stimulated with LPS promoted FOXO3 acetylation and ubiquitinoylation. FOXO3 K241, K258, K270, K289, and K568 are potential SIRT3-targeted acetylation sites on FOXO3 in mice (Supplementary Table 2)^39^. We further determined these potential deacetylation sites. TGR5KO BMDMs were transfected with individual site-specific mutant plasmids before stimulated with LPS. The acetylation levels of FOXO3 were examined. The result suggested that only the K270Q and K289Q plasmids transfection significantly decreased the FOXO3 acetylation level (Fig. 6c). To further determine the interaction between post-translational modifications (PTMs) in FOXO3 subcellular localization, protein levels, DNA-binding properties and transcriptional activity, we overexpressed SIRT3 or knockdown it. The results demonstrated that neither SIRT3 overexpression (Fig. 6d) nor SIRT3 knockdown (Fig. 6e) changed FOXO3 acetylation level in BMDMs transfected with K270/289R (acetylation mimetic mutant) or K270/289Q (deacetylation mimetic mutant) plasmid, which suggested that K270 and K289 might be the main targets of SIRT3 on FOXO3 in BMDMs stimulated with LPS. To further explore the mechanisms, TGR5KO BMDMs were transfected with FOXO3WT, FOXO3K270R, FOXO3K270Q, FOXO3K289R, FOXO3K289Q, FOXO3K270/289R, or FOXO3K270/289Q mutant plasmids and then stimulated with LPS. The results showed that lysine to arginine substitutions at Lys270 and Lys289 substantially repressed FOXO3 ubiquitination and degradation (Fig. 6f). Furthermore, FOXO3 overexpression decreased ROS levels in TGR5KO BMDMs (Fig. 6g). Meanwhile, FOXO3 overexpression reduced proinflammatory TNF-α and IL-6 expression and promoted anti-inflammatory IL-10 expression in the supernatant of TGR5KO BMDMs treated with LPS (Fig. 6h). Together, these results demonstrated that SIRT3 regulated macrophage oxidative stress and inflammatory response by inhibiting the acetylation and ubiquitination of FOXO3.

### FOXO3 regulates macrophage oxidative stress and inflammatory response via inhibiting HIF-1α transcription activity

**Fig. 7:**
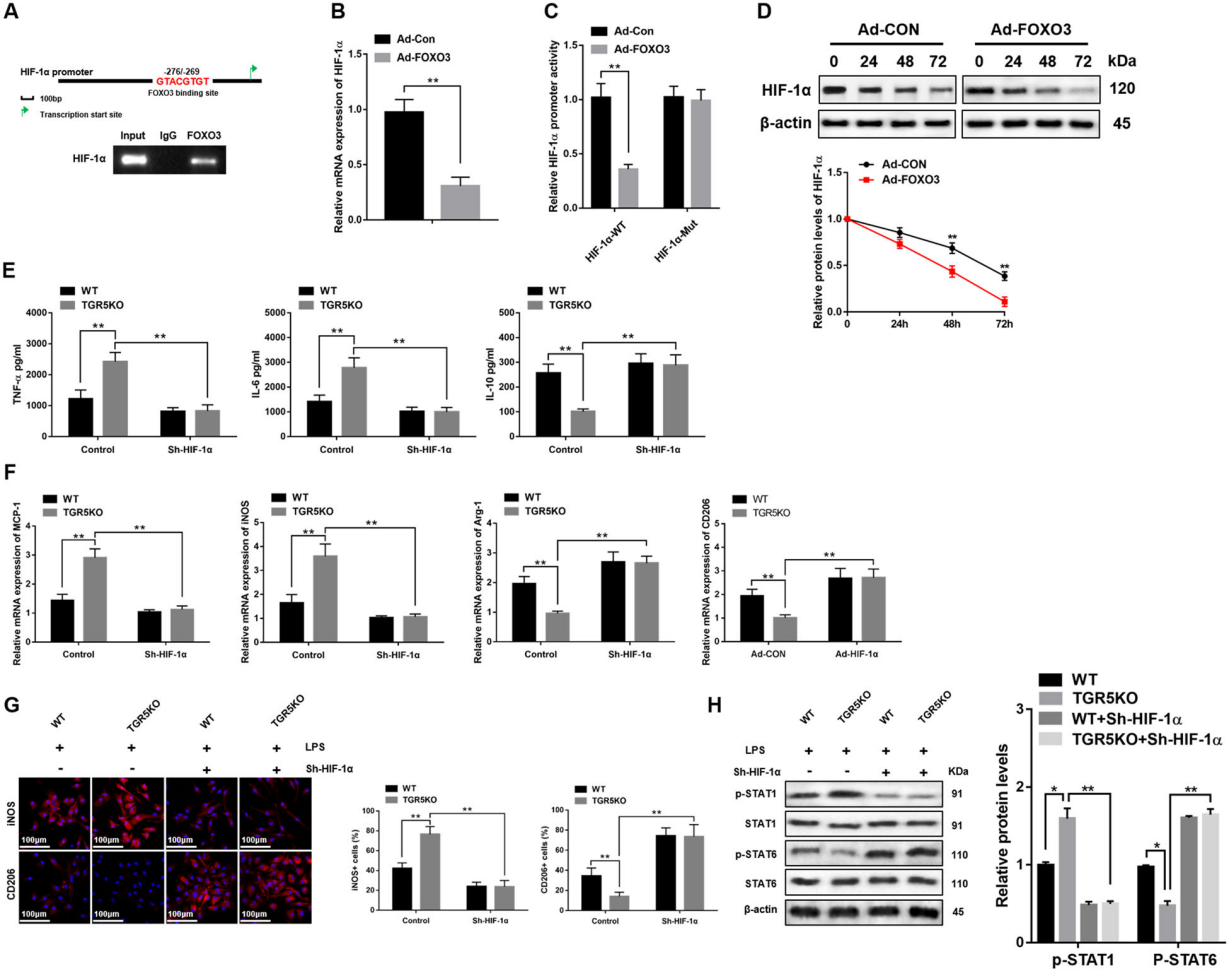
FOXO3 regulates macrophage oxidative stress and inflammatory response via inhibiting HIF-1α transcription activity. **a** Illustrating diagram showed the potential site that FOXO3 bound with the HIF-1α promoter in mouse. BMDMs transfected with Ad-FOXO3 was performed with ChIP assay with anti-FOXO3 or IgG antibody. Representative of three independent experiments. **b** The expression of HIF-1α in LPS-stimulated BMDMs transfected with Ad-FOXO3 or Ad-CON was detected by qRT-PCR, *n* = 3/group. **c** HIF-1α promoter (HIF-1α-WT) or the mutant of HIF-1α promoter (HIF-1α-Mut) luciferase reporter activity was detected in LPS-stimulated BMDMs infected with Ad-FOXO3 or Ad-CON. **d** Western blots were carried out to detect the level of HIF-1α in LPS-stimulated BMDMs infected with Ad-FOXO3 or Ad-CON. Representative of three experiments. **e** Inflammatory gene expression in supernatant from LPS-stimulated BMDMs infected with HIF-1α siRNA or control was measured by ELISA. *n* = 3/group. **f** M1 markers (MCP-1 and iNOS) and M2 markers (Arg-1 and CD206) of gene induction in LPS-stimulated BMDMs infected with HIF-1α siRNA or control were detected by qRT-PCR. *n* = 3/group. **g** Immunofluorescence was performed to detect iNOS+ and CD206+ cells in LPS-stimulated BMDMs infected with HIF-1α siRNA or control (original magnification ×200). Nuclear was stained with DAPI. Representative of three experiments. Ratio of iNOS+ and CD206+ cells in different experimental groups. *n* = 3/group. **h** Western blots were performed to detect intracellular p-STAT1, p-STAT6, and β-actin protein levels in LPS-stimulated BMDMs infected with HIF-1α siRNA or control. Representative of three experiments. Mean ± SEM; **P < 0.01; *P < 0.05.

To further explore how FOXO3 regulated macrophage immune response, a potential site that FOXO3 bound with HIF-1α promoter (−276/−269) in mouse was uncovered by bioinformatics analysis and confirmed by ChIP assays (Fig. 7a). The result suggested that overexpression of FOXO3 decreased HIF-1α mRNA expression in WT BMDMs treated with LPS (Fig. 7b). Moreover, the mutation of FOXO3 binding site within HIF-1α promoter eliminated the inhibition of HIF-1α promoter activity induced by FOXO3 overexpression in WT BMDMs (Fig. 7c). Next, FOXO3 overexpression decreased HIF-1α protein expression in a time-dependent manner (Fig. 7d). To further determine the role of HIF-1α in TGR5KO BMDMs, siRNA was used to knockdown HIF-1α. The results indicated that HIF-1α knockdown reduced proinflammatory TNF-α and IL-6 expression and promoted anti-inflammatory IL-10 expression in the supernatant of TGR5KO BMDMs treated with LPS (Fig. 7e). Moreover, HIF-1α knockdown markedly depressed MCP-1 and iNOS expression but increased Arg-1 and CD206 expression (Fig. 7f). Immunofluorescence suggested that HIF-1α knockdown significantly decreased the number of iNOS (M1 marker) positive cells in TGR5KO BMDMs treated with LPS, but increased the number of CD206 (M2 marker) positive cells in TGR5KO BMDMs treated with LPS (Fig. 7g). Furthermore, western blot showed HIF-1α knockdown markedly inhibited STAT1 activation but promoted STAT6 activation in TGR5KO BMDMs treated with LPS (Fig. 7h).

### **Myeloid TGR5** alleviates hepatic ischemia/reperfusion injury

**Fig. 8:**
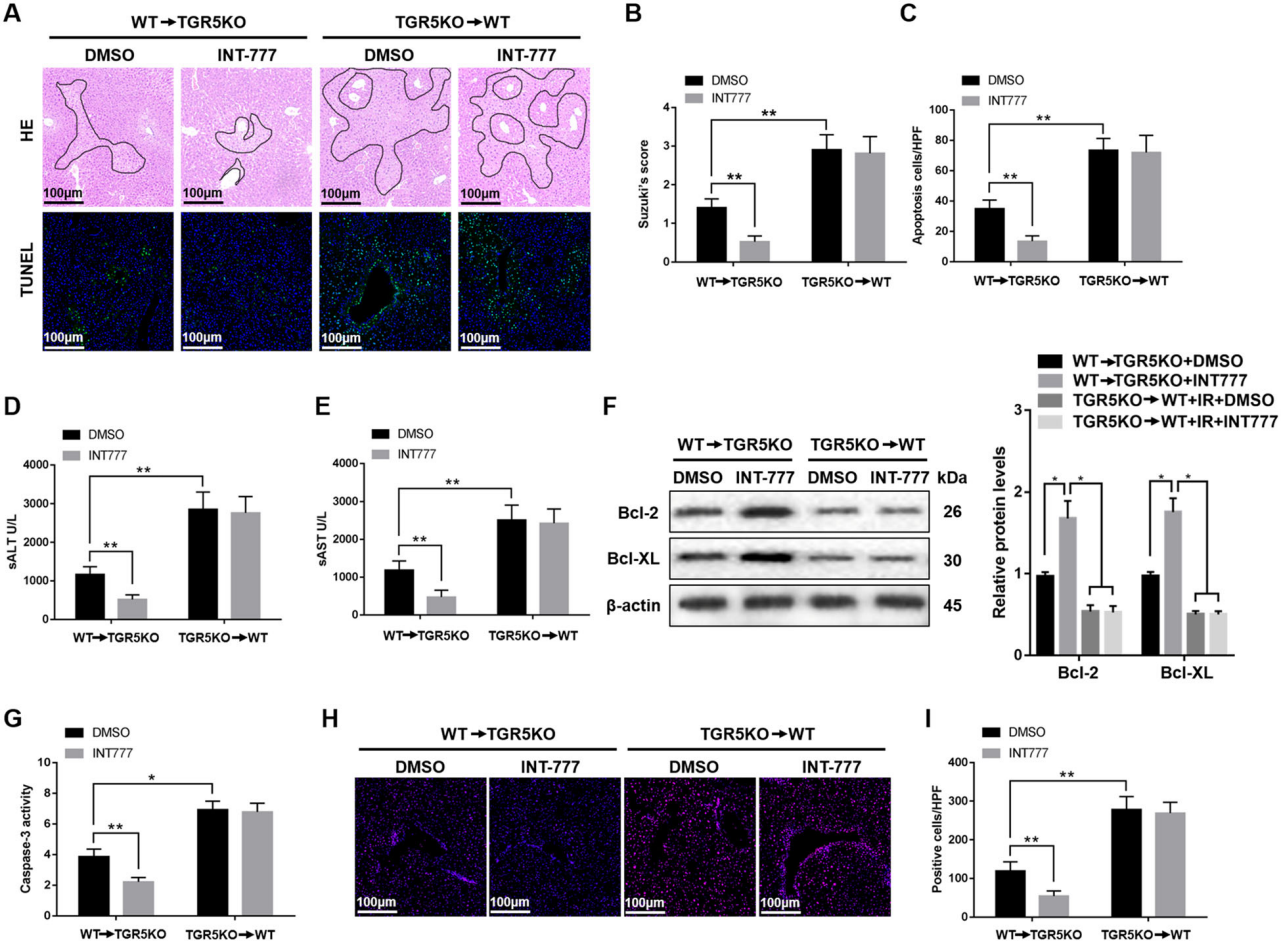
Myeloid TGR5 alleviates hepatic ischemia/reperfusion injury. WT → TGRKO and TGR5KO → WT chimeric mice were performed with 90 min of warm ischemia followed by 6 h of reperfusion and INT-777 were administered 2 days prior to the liver ischemia, as described in “Materials and methods” section. **a** Livers harvested after 6 h reperfusion were subjected to histopathology. Liver sections were performed with TUNEL staining (original magnification ×200). Nuclear was stained with DAPI. Representative of six mice/group. **b** The damage of liver architecture was evaluated by Suzuki’s score. *n* = 6 mice/group. **c** TUNEL-positive cells were quantified in different experimental groups (original magnification ×200). *n* = 6 mice/group. **d**, **e** Hepatocellular function was detected by serum ALT (U/L) and AST (U/L). *n* = 6 mice/group. **f** Western blots were performed to analyze Bcl-2 and Bcl-XL protein levels in liver tissues. Representative of three experiments. **g** Caspase-3 activity assay was performed to determine cellular activity. *n* = 6 mice/group. **h**, **i** The levels of ROS in liver tissues was examined by DHE (original magnification ×200). Representative of six mice/group. Cells labeled with red fluorescent were counted blindly in 10 HPF/section (original magnification ×200). The numbers of ROS-producing cells (red) per high power field were quantified (original magnification ×200), *n* = 6 mice/group. Mean ± SEM; **P < 0.01; *P < 0.05.

To investigate the effect of myeloid-derived TGR5 during liver IR injury, the chimeric mice were generated and subjected to 90 min of warm ischemia followed by 6 h of reperfusion. Figure 8a–c showed that reduced liver architecture damage and hepatocyte apoptosis were found in WT → TGR5KO group compared to the TGR5KO → WT group. TGR5 activation with INT-777 ameliorated hepatic IR injury in WT → TGR5KO group compared to the TGR5KO → WT group (Fig. 8a–c), as evidenced by serum ALT and AST (Fig. 8d, e), as well as the levels of anti-apoptotic proteins (Bcl-2 and Bcl-XL) expression (Fig. 8f) and caspase-3 activity (Fig. 8g). Moreover, the level of ROS in WT → TGR5KO group treated with INT-777 was markedly decreased compared to the TGR5KO → WT group treated with INT-777 (Fig. 8h, i). Collectively, these results indicated that TGR5 in myeloid cells played a crucial role in alleviating hepatic IR injury.

## Discussion

This study verified the molecular mechanism of TGR5 affecting hepatic I/R injury via SIRT3/FOXO3/HIF-1α pathway. TGR5 knockout inhibited SIRT3 expression. The effect of SIRT3 deacetylating FOXO3 was impaired at lysine 270 and 289. The acetylation, ubiquitination and degradation of FOXO3 increased. The effect of FOXO3 inhibiting HIF-1α transcription by binding to its promoter was decreased. The expression levels of ROS and inflammatory cytokines increased. TGR5 activation reversed above results.

TGR5 (also known as GPBAR1), a member of the G-protein-coupled receptor family, contains seven transmembrane domains and transduces extracellular signals via heterotrimeric G proteins. TGR5 can maintain energy homeostasis and bile acid homeostasis^40^. A previous study demonstrated that TGR5 activation inhibited oxidative stress and induced mitochondrial biogenesis by promoting SIRT1, SIRT3, and Nrf-1 expression, indicating a protective role for TGR5 inhibiting obesity-related and diabetes-related kidney disease^12^. Moreover, studies indicated that TGR5 activation induced potent anti-inflammatory effects by inhibiting nuclear translocation of nuclear factor kappa B and suppressing cytokine production in hepatic macrophages^41,42^. However, the role and mechanism of TGR5 regulating hepatic I/R injury remains largely unknown. Our study demonstrated that TGR5 deficiency exacerbated hepatic I/R injury by inhibiting SIRT3 expression.

SIRT3, a member of sirtuin family, can regulate oxidative stress potently^43,44^. Studies demonstrated that SIRT3 might modulate the acetylation of NADH dehydrogenase 1 alpha subcomplex subunit 9 and succinate dehydrogenase complex, as well as mitochondrial oxidative stress^45–47^. Study indicated that SIRT3 could target some proteins during I/R, including manganese superoxide dismutase, isocitrate dehydrogenase 2 and ect^48^. FOXO3, a potential SIRT3 target, belongs to the forkhead box class O transcription factor family which can regulate various cellular and physiological processes, such as metabolism, development, and tumor suppression^49–51^. A previous study indicated that SIRT3 deacetylated FOXO3 and stabilized its expression. This process enhanced the mitochondrial antioxidant system and promoted ECs adaptive to hypoxia^52^. Another study showed that SIRT3 deacetylating FOXO3 was induced by hydrogen peroxide, and a set of genes vital to mitochondrial homeostasis were upregulated^53^. Our study demonstrated that SIRT3 deacetylated FOXO3 to protect murine hepatic I/R. We found that deacetylation of FOXO3 at lysine 270 and 289 inhibited FOXO3 ubiquitination and degradation. Deacetylation of FOXO3 suppressed macrophages ROS levels and inflammatory response.

HIF-1 is an essential regulator of oxygen homeostasis^54^. As a heterodimer, HIF-1 consists of inducibly expressed HIF-1α and constitutively expressed HIF-1β subunits, binding to the specific DNA sequences regarded as hypoxia response elements (HREs). The regulation of the synthesis, degradation, and transactivation function of the HIF-1α subunit determined the biologic activity of HIF-1^55^. Hypoxia promotes HIF-1α expression in rheumatoid arthritis (RA), which is reported to induce macrophages M1 subtype polarization^56–59^ by increasing ROS production^60^. The inhibition of HIF-1α can inhibit M1 phenotype and promote macrophages M2 polarization^61^, and effectively relieve RA^32,62–64^. The mechanisms of HIF-1 protein levels and transcriptional activity regulation have been extensively studied. Studies demonstrated that SIRT3 could repress tumor growth by downregulating HIF-1α and decreasing ROS accumulation to regulate the Warburg effect in cancer cells and inhibit tumorigenesis^25,26,65^. Another study showed that FOXO3 inhibited HIF-1α and decreased ROS accumulation to reduce HIF-1α-induced apoptosis during hypoxia^35^. Moreover, study indicated that FOXO3 overexpression downregulated HIF-1α protein levels and inhibited HIF-1α transcription in renal I/R injury^66^. Our study demonstrated that HIF-1α promoted macrophages ROS levels and inflammatory response. FOXO3 overexpression inhibited HIF-1α transcription and repressed macrophages inflammation in hepatic I/R injury.

Taken together, this was the first study to disclose that TGR5 deficiency aggravated hepatic I/R injury by inhibiting SIRT3 expression, promoting HIF-1α transcription, ROS levels, and inflammatory response in macrophages via increasing acetylation, ubiquitination and degradation of FOXO3. Our study demonstrated that TGR5 and SIRT3/FOXO3/ HIF-1α axis may serve as therapeutic targets for hepatic I/R injury.

## Materials and methods

### **A**nimals

Eight-week-old C57BL/6J wild-type (WT) male mice were purchased from the Laboratory Animal Resources Center of Nanjing Medical University. TGR5 knockout (TGR5KO) male mice (C57BL/6J background) were offered by Johan Auwerx and Kristina Schoonjans. All mice were fed under specific pathogen-free conditions. All animal experiments were performed according to the recommendations in the protocol (number NMU08-092), which was approved by the Institutional Animal Care and Use Committee of Nanjing Medical University.

### Experimental model

This study used a well-established mouse model of segmental (70%) hepatic warm IR injury. Briefly, mice were injected with 10% chloral hydrate intraperitoneally (0.3 g/kg) and a midline laparotomy was performed. Mice were injected with heparin (100 U/kg). An atraumatic bulldog clamp was used to interrupt all structures in the portal triad (hepatic artery, portal vein, and bile duct). The clip was removed for reperfusion after 90 min of ischemia. The mice were sacrificed at 6 h after reperfusion. Blood and liver tissue samples were collected for further analysis. Animals were randomly divided into WT sham group, TGR5KO sham group, WT IR group, and TGR5KO IR group. In the sham group (*n* = 6), mice were subjected to laparotomy without hepatic ischemia. In the WT IR group (*n* = 6) and TGR5KO IR group (*n* = 6), mice were subjected to IR as described above. In some experiments, mice were fed with INT-777 for 2 days (30 mg/kg/day) prior to IR.

### Histopathological analysis

Liver specimens were fixed in 10% buffered formalin and embedded in paraffin. Sections of liver tissues (4 μm) were stained with hematoxylin and eosin (HE). The severity of liver IR injury was graded using Suzuki’s criteria on a scale from 0 to 4.

### Hepatocellular function analysis

Blood samples were centrifuged to obtain serum. Automated chemical analyzer (Olympus, Tokyo, Japan) was used to detect the levels of serum alanine transaminase (ALT) and aspartate transaminase (AST).

### TUNEL fluorescent assay

TUNEL staining was conducted with an in situ fluorescence detection kit (Roche, Basel, Switzerland) according to the manufacturer’s protocols using freshly frozen tissues. Sections of liver tissues were counterstained by 4′,6-diamidino-2-phenylindole (DAPI) (Beyotime, shanghai, China) for 5 min. TUNEL-positive cells numbers in liver tissues were counted and analyzed (×200 magnification).

### Caspase-3 activity analysis

Caspase-3 activity was conducted and detected by an assay kit (Calbiochem, La Jolla, CA) in accordance with the manufacturer’s protocols. Briefly, liver tissues were resuspended in lysis buffer containing 1 mmol/L DTT, 0.1% CHAPS, 50 mmol/L HEPES, 0.1% Triton X-100, and 0.1 mmol/L EDTA, and then were incubated for 30 min on ice. Lysate was centrifuged at 16,000 × *g* for 10 min at 4 °C. The supernatants were incubated with 200 μM of enzyme-specific colorimetric caspase-3 substrate for 2 h at 37 °C. Caspase-3 activity was analyzed by detecting the absorbance at 405 nm wavelength.

### Determination of oxidative stress

Freshly frozen tissues were dyed with dihydroethidium (DHE) (beyotime, shanghai, China), dyed for nucleus with DAPI (beyotime, shanghai, China), and observed by fluorescence microscope (Olympus, Tokyo, Japan) in accordance to the manufacturer’s instructions. The amount of ROS-positive cells were counted (×200 magnification). To assess the cellular ROS level, BMDMs were incubated with 2′,7′-dichlorodihydrofluorescein diacetate (DCFH-DA) (beyotime, shanghai, China) for 0.5 h (37 °C). The fluorescence intensity of ROS was measured using confocal microscopy (ZEISS, Oberkochen, Germany) according to the manufacturer’s instructions.

### **B**one marrow transplantation

Cesium source was used to irradiate male WT mice (5-week-old) twice with a dose of 550 rad (5.5 Gy) 4 h before transplantation. Bone marrow was isolated from femurs of WT or TGR5KO mice by flushing with sterile Opti-MEM (Thermo Fisher Scientific, MA, USA). 1 × 10^7^ bone marrow cells was injected into each recipient mouse through retro-orbital injection. Bone marrow transplantations were performed to generate the chimeric mice as follows: WT → TGR5KO (expressing TGR5 only in myeloid cells); TGR5KO → WT (expressing TGR5 only in non-myeloid cells). Four weeks after bone marrow transplantation, the chimeras were subjected to a liver IR injury model. Chimeras were treated with clodronate-containing liposomes 48 h prior to the liver ischemia to delete tissue-resident macrophages^36^.

### Cell culture and treatment

M-CSF (20 ng/mL) (Sino Biologic, Beijing, China) was used to generate bone marrow-derived macrophages (BMDMs) in vitro as previously described^5^. BMDMs were cultured in DMEM supplemented with 10% fetal calf serum. The negative control (sc-37007) and specific siRNAs including SIRT3 siRNA (sc-61556), and HIF-1α siRNA (sc-35562) were purchased (Santa Cruz Biotechnology, CA, USA). BMDMs transfection was conducted with Lipofectamine 2000 (Invitrogen, Carlsbad, CA, USA) according to the manufacturer’s specifications. Adenoviruses (GenePharma, Shanghai, China) expressing SIRT3, FOXO3, and Vector (designated as Ad-SIRT3, Ad-FOXO3, and Ad-CON) were used to transfect BMDMs. After 48 h, cells were supplied with 100 ng/mL of LPS (Sigma, St. Louis, MO, USA) for additional 6 h, which is a major component of the outer membrane of Gram-negative bacteria and a potent activator of macrophages in vitro to simulate injury and inflammation in vivo^13^.

### Plasmid construction and transfection

The FOXO3 mutant plasmids were synthesized (GenePharma, Shanghai, China). Two microgram of the FOXO3 mutant plasmids or the control vector were transfected into BMDMs with Lipofectamine 2000 (Invitrogen, Carlsbad, CA, USA) for 48 h. BMDMs were subjected to 100 ng/mL of LPS for additional 6 h. The transfection procedures were performed following the manufacturer’s protocols.

### Western blot assay

Proteins were extracted from liver tissues and cells with ice-cold lysis buffer (50 mM Tris, 150 mM Nacl, 1% sodium deoxycholate, 0.1% sodium dodecyl sulfate, and 1% Triton-100). The following primary antibodies were used to incubate membranes: Bcl-2, Bcl-XL, SIRT3, FOXO3, HIF-1α, p-STAT1, STAT1, p-STAT6, STAT6, β-actin rabbit mAbs (Cell Signaling Technology, MA, USA), and TGR5 rabbit pAb (Abcam, Cambridge, UK), FOXO3 mouse mAb (Proteintech, Wuhan, China). The reactions were detected with HRP-conjugated goat anti-rabbit immunoglobulin G (IgG) (Cell Signaling Technology, MA, USA) or goat anti-mouse IgG (H + L) secondary antibodies.

### Real-time qPCR analysis

Total RNA was purified from the liver tissues or BMDMs with TRIzol reagent (Invitrogen, Carlsbad, CA, USA) and using a cDNA Synthesis Kit (Roche, Indianapolis, IN, USA) to perform cDNA reverse transcription. The target genes were amplified with a LightCycler 480 SYBR Green 1 Master Mix (Roche, Indianapolis, IN, USA). Quantitative realtime-PCRs were all repeated three times. The results were uniformly normalized to β-actin. The primers used in our study are presented in Supplementary Table 1.

### **Enzyme-linked immunosorbent assay** (ELISA)

The quantifications of TNF-α, IL-6, and IL-10 (Abcam, Cambridge, UK) levels in serum or cell culture supernatants were performed with an ELISA kit following the manufacturer’s protocols.

### Immunohistochemical and immunofluorescence staining

Tissues fixed with 4% formalin were embedded in paraffin. Then, tissue sections were incubated with primary antibody of SIRT3 (Invitrogen, Carlsbad, CA, USA) or FOXO3 (Proteintech, Wuhan, China). Biotinylated goat antirat IgG (Vector, Burlingame, CA, USA), as the secondary antibody, was incubated with immunoperoxidase (ABC Kit, Vector) following the manufacturer’s protocols. SIRT3 and FOXO3 in BMDMs were detected by immunofluorescence with antirabbit SIRT3 pAb (Invitrogen, Carlsbad, CA, USA) and antimouse FOXO3 mAb (Proteintech, Wuhan, China), followed by incubation with secondary goat antirabbit Texas Green-conjugated IgG or goat antimouse Texas Red-conjugated IgG (Sigma, St. Louis, MO, USA). DAPI was used to stain the nuclei. The slides were washed twice with PBS and detected using confocal microscopy (ZEISS, Oberkochen, Germany) in accordance to the manufacturer’s protocols. Positive cells were observed and counted in 10 HPF/section (×200 or ×400).

### Immunoprecipitation and ubiquitination analysis

BCA protein concentration assay kit (Thermo Fisher Scientific, MA, USA) was used to detect the protein concentration of BMDMs. One milligram of total protein lysates from each sample were used to perform immunoprecipitation (IP), and then were incubated with rabbit polyclonal IgG control antibody, rabbit monoclonal anti-SIRT3 (Abcam, Cambridge, UK) or mouse monoclonal anti-FOXO3 (Proteintech, Wuhan, China). Then, the lysates were rotated for 4 h (4 °C). Subsequently, 25 μL protein A/G PLUS-Agarose was added to resuspend the lysates and the mixture continues to rotate for another 2 h. The eluted proteins were immunoblotted with the rabbit monoclonal anti-SIRT3 (Abcam, Cambridge, UK) or mouse monoclonal antiFOXO3 (Proteintech, Wuhan, China) after washing and denaturing with immunoprecipitation buffer. For the acetylation and ubiquitination analysis, the BMDMs were infected with FOXO3 mutant plasmids with effectene transfection reagent (Qiagen, Hilden, Germany) for 48 h, stimulated with LPS for 6 h, and then incubated with carbobenzoxy-Leu-Leu-leucinal (MG132) (Sigma, St.Louis, MO, USA) for 4 h. The lysates were incubated with antiFOXO3 overnight, and then added with protein A/G beads (Roche, Basel, Switzerland) for 2 h. The precipitates were boiled for 5 mins and were detected with antibodies against Acetyl lysine (Abcam, Cambridge, UK), Ubiquitin, and β-actin (Cell Signaling Technology, MA, USA) by immunoblot.

### **Chromatin immunoprecipitation assays** (ChIP)

ChIP assay was performed with Magna ChIP HiSens Kit (Millipore, Bedford, Massachusetts, USA) according to the manufacturer’s protocols. The chromatin was immunoprecipitated with antiIgG or antiFOXO3 antibody (Abcam, Cambridge, UK). The DNA was purified and the bound sequences were analyzed by PCR. The primers were as follows: HIF-1α, forward: 5′-AGACGGGTTGGATTGAAAG-3′, reverse: 5′-GACTTACAGGAGGCAAACA-3′.

### Statistical analysis

All data were presented as the mean ± SEM and were analyzed using SPSS22.0 software. Student’s unpaired *t*-test and one-way ANOVA were used to analyze differences among different groups. Statistical significance was regarded as *P* < 0.05 (* for difference less than *P* < 0.05, ** for difference less than *P* < 0.01).

## Supplementary information

Supplementary Table 1

Supplementary Table 2

## References

[1] Lu L (2016). Innate immune regulations and liver ischemia-reperfusion injury. Transplantation.

[2] Rubartelli A, Lotze MT (2007). Inside, outside, upside down: damage-associated molecular-pattern molecules (DAMPs) and redox. Trends Immunol..

[3] Lotze MT (2007). The grateful dead: damage-associated molecular pattern molecules and reduction/oxidation regulate immunity. Immunol. Rev..

[4] Huang H (2015). Damage-associated molecular pattern-activated neutrophil extracellular trap exacerbates sterile inflammatory liver injury. Hepatology.

[5] Lu L (2018). Myeloid Notch1 deficiency activates the RhoA/ROCK pathway and aggravates hepatocellular damage in mouse ischemic livers. Hepatology.

[6] Keitel V (2007). The G-protein coupled bile salt receptor TGR5 is expressed in liver sinusoidal endothelial cells. Hepatology.

[7] Keitel V, Donner M, Winandy S, Kubitz R, Häussinger D (2008). Expression and function of the bile acid receptor TGR5 in Kupffer cells. Biochem Biophys. Res Commun..

[8] Keitel V (2009). The membrane-bound bile acid receptor TGR5 is localized in the epithelium of human gallbladders. Hepatology.

[9] Thomas C, Auwerx J, Schoonjans K (2008). Bile acids and the membrane bile acid receptor TGR5-connecting nutrition and metabolism. Thyroid.

[10] Thomas C (2009). TGR5-mediated bile acid sensing controls glucose homeostasis. Cell Metab..

[11] Broeders EP (2015). The bile acid chenodeoxycholic acid increases human brown adipose tissue activity. Cell Metab..

[12] Wang XX (2016). G protein-coupled bile acid receptor TGR5 activation inhibits kidney disease in obesity and diabetes. J. Am. Soc. Nephrol..

[13] Wang YD, Chen WD, Yu D, Forman BM, Huang W (2011). The G-protein-coupled bile acid receptor, Gpbar1 (TGR5), negatively regulates hepatic inflammatory response through antagonizing nuclear factor κ light-chain enhancer of activated B cells (NF-κB) in mice. Hepatology.

[14] Haselow K (2013). Bile acids PKA-dependently induce a switch of the IL-10/IL-12 ratio and reduce proinflammatory capability of human macrophages. J. Leukoc. Biol..

[15] Yoneno K (2013). TGR5 signalling inhibits the production of pro-inflammatory cytokines by in vitro differentiated inflammatory and intestinal macrophages in Crohn’s disease. Immunology.

[16] Hallows WC (2011). Sirt3 promotes the urea cycle and fatty acid oxidation during dietary restriction. Mol. Cell.

[17] Hirschey MD (2010). SIRT3 regulates mitochondrial fatty-acid oxidation by reversible enzyme deacetylation. Nature.

[18] Shimazu T (2010). SIRT3 deacetylates mitochondrial 3-hydroxy-3-methylglutaryl CoA synthase 2 and regulates ketone body production. Cell Metab..

[19] Wang Z (2020). SIRT3-mediated deacetylation of PRDX3 alleviates mitochondrial oxidative damage and apoptosis induced by intestinal ischemia/reperfusion injury. Redox Biol..

[20] Hebert AS (2013). Calorie restriction and SIRT3 trigger global reprogramming of the mitochondrial protein acetylome. Mol. Cell.

[21] Chang G, Chen Y, Zhang H, Zhou W (2019). Trans sodium crocetinate alleviates ischemia/reperfusion-induced myocardial oxidative stress and apoptosis via the SIRT3/FOXO3a/SOD2 signaling pathway. Int. Immunopharmacol..

[22] Bochaton T (2015). Inhibition of myocardial reperfusion injury by ischemic postconditioning requires sirtuin 3-mediated deacetylation of cyclophilin D. J. Mol. Cell Cardiol..

[23] Pi H (2015). SIRT3-SOD2-mROS-dependent autophagy in cadmium-induced hepatotoxicity and salvage by melatonin. Autophagy.

[24] Liu L (2019). Melatonin ameliorates cerebral ischemia/reperfusion injury through SIRT3 activation. Life Sci..

[25] Finley LW (2011). SIRT3 opposes reprogramming of cancer cell metabolism through HIF1α destabilization. Cancer Cell.

[26] Bell EL, Emerling BM, Ricoult SJ, Guarente L (2011). SirT3 suppresses hypoxia inducible factor 1α and tumor growth by inhibiting mitochondrial ROS production. Oncogene.

[27] Katwal G (2018). SIRT3 a major player in attenuation of hepatic ischemia-reperfusion injury by reducing ROS via its downstream mediators: SOD2, CYP-D, and HIF-1α. Oxid. Med Cell Longev..

[28] Zhao H (2018). Sirt3 inhibits cerebral ischemia-reperfusion injury through normalizing Wnt/β-catenin pathway and blocking mitochondrial fission. Cell Stress Chaperones.

[29] Zhai M (2017). Melatonin ameliorates myocardial ischemia reperfusion injury through SIRT3-dependent regulation of oxidative stress and apoptosis. J Pineal Res..

[30] Lee FY (2018). Daily melatonin protects the endothelial lineage and functional integrity against the aging process, oxidative stress, and toxic environment and restores blood flow in critical limb ischemia area in mice. J. Pineal Res..

[31] Semenza GL (2007). Life with oxygen. Science.

[32] Cramer T (2003). HIF-1alpha is essential for myeloid cell-mediated inflammation. Cell.

[33] Zhang XL (2011). Activation of hypoxia-inducible factor-1 ameliorates postischemic renal injury via inducible nitric oxide synthase. Mol. Cell Biochem..

[34] Ferber EC (2012). FOXO3a regulates reactive oxygen metabolism by inhibiting mitochondrial gene expression. Cell Death Differ..

[35] Bakker WJ, Harris IS, Mak TW (2007). FOXO3a is activated in response to hypoxic stress and inhibits HIF1-induced apoptosis via regulation of CITED2. Mol. Cell.

[36] Yue S (2017). Prolonged ischemia triggers necrotic depletion of tissue-resident macrophages to facilitate inflammatory immune activation in liver ischemia reperfusion injury. J. Immunol..

[37] Sundaresan NR (2009). Sirt3 blocks the cardiac hypertrophic response by augmenting Foxo3a-dependent antioxidant defense mechanisms in mice. J. Clin. Invest..

[38] Jacobs KM (2008). SIRT3 interacts with the daf-16 homolog FOXO3a in the mitochondria, as well as increases FOXO3a dependent gene expression. Int. J. Biol. Sci..

[39] Brunet A (2004). Stress-dependent regulation of FOXO transcription factors by the SIRT1 deacetylase. Science.

[40] Donepudi AC, Boehme S, Li F, Chiang JY (2017). G-protein-coupled bile acid receptor plays a key role in bile acid metabolism and fasting-induced hepatic steatosis in mice. Hepatology.

[41] Schaap FG, Trauner M, Jansen PL (2014). Bile acid receptors as targets for drug development. Nat. Rev. Gastroenterol. Hepatol..

[42] Perino A, Schoonjans K (2015). TGR5 and immunometabolism: insights from physiology and pharmacology. Trends Pharm. Sci..

[43] Cornelius C (2013). Cellular stress response, sirtuins and UCP proteins in Alzheimer disease: role of vitagenes. Immun. Ageing.

[44] Bagul PK, Banerjee SK (2013). Insulin resistance, oxidative stress and cardiovascular complications: role of sirtuins. Curr. Pharm. Des..

[45] Ahn BH (2008). A role for the mitochondrial deacetylase Sirt3 in regulating energy homeostasis. Proc. Natl Acad. Sci. USA.

[46] Finley LW (2011). Succinate dehydrogenase is a direct target of sirtuin 3 deacetylase activity. PLoS ONE.

[47] Lombard DB (2007). Mammalian Sir2 homolog SIRT3 regulates global mitochondrial lysine acetylation. Mol. Cell Biol..

[48] Porter GA, Urciuoli WR, Brookes PS, Nadtochiy SM (2014). SIRT3 deficiency exacerbates ischemia-reperfusion injury: implication for aged hearts. Am. J. Physiol. Heart Circ. Physiol..

[49] van der Horst A, Burgering BM (2007). Stressing the role of FoxO proteins in lifespan and disease. Nat. Rev. Mol. Cell Biol..

[50] Calnan DR, Brunet A (2008). The FoxO code. Oncogene.

[51] Daitoku H, Sakamaki J, Fukamizu A (2011). Regulation of FoxO transcription factors by acetylation and protein–protein interactions. Biochim Biophys. Acta.

[52] Tseng AH, Wu LH, Shieh SS, Wang DL (2014). SIRT3 interactions with FOXO3 acetylation, phosphorylation and ubiquitinylation mediate endothelial cell responses to hypoxia. Biochem. J..

[53] Tseng AH, Shieh SS, Wang DL (2013). SIRT3 deacetylates FOXO3 to protect mitochondria against oxidative damage. Free Radic. Biol. Med..

[54] Semenza GL (1999). Regulation of mammalian O2 homeostasis by hypoxia-inducible factor 1. Annu. Rev. Cell Dev. Biol..

[55] Wang GL, Jiang BH, Rue EA, Semenza GL (1995). Hypoxia-inducible factor 1 is a basic-helix-loop-helix-PAS heterodimer regulated by cellular O2 tension. Proc. Natl Acad. Sci. USA.

[56] Wang T (2017). HIF1α-induced glycolysis metabolism is essential to the activation of inflammatory macrophages. Mediators Inflamm..

[57] Lan G (2018). Nanoscale metal-organic framework overcomes hypoxia for photodynamic therapy primed cancer immunotherapy. J. Am. Chem. Soc..

[58] Xu R (2016). Nanoscale metal-organic frameworks for ratiometric oxygen sensing in live cells. J. Am. Chem. Soc..

[59] Sun X, Niu G, Chan N, Shen B, Chen X (2011). Tumor hypoxia imaging. Mol. Imaging Biol..

[60] Fearon U, Canavan M, Biniecka M, Veale DJ (2016). Hypoxia, mitochondrial dysfunction and synovial invasiveness in rheumatoid arthritis. Nat. Rev. Rheumatol..

[61] Werno C (2010). Knockout of HIF-1α in tumor-associated macrophages enhances M2 polarization and attenuates their pro-angiogenic responses. Carcinogenesis.

[62] Manabe H (2008). Inhibition of histone deacetylase down-regulates the expression of hypoxia-induced vascular endothelial growth factor by rheumatoid synovial fibroblasts. Inflamm. Res..

[63] Shankar J, Thippegowda PB, Kanum SA (2009). Inhibition of HIF-1alpha activity by BP-1 ameliorates adjuvant induced arthritis in rats. Biochem. Biophys. Res. Commun..

[64] Kim J (2019). Synergistic oxygen generation and reactive oxygen species scavenging by manganese ferrite/ceria co-decorated nanoparticles for rheumatoid arthritis treatment. ACS Nano..

[65] Schumacker PT (2011). SIRT3 controls cancer metabolic reprogramming by regulating ROS and HIF. Cancer Cell.

[66] Xie Y (2018). Ischemic preconditioning attenuates ischemia/reperfusion-induced kidney injury by activating autophagy via the SGK1 signaling pathway. Cell Death Dis..

