# Supplementary material for: TGR5 deficiency aggravates hepatic ischemic/reperfusion injury via inhibiting SIRT3/FOXO3/HIF-1ɑ pathway
Source: Cell Death Discov. 2020 Nov 1;6:116. doi: 10.1038/s41420-020-00347-2 (PMC7604280; doi:10.1038/s41420-020-00347-2)
Supplement: Supplementary file 1 — Supplementary Table 1 [file 41420_2020_347_MOESM1_ESM.docx]

**Supplementary Table 1: Primer sequences for the amplification**.

Gene Forward Primer (5′ → 3′) Reverse Primer (5′ → 3′)

TGR5 5’-CCTGGCAAGCCTCATCGTC-3’ 5’-AGCAGCCCGGCTAGTAGTAG-3’

TNF-α 5’-GACGTGGAACTGGCAGAAGAG-3’ 5’-TTGGTGGTTTGTGAGTGTGAG-3’

IL-6 5’-CCAAGAGGTGAGTGCTTCCC-3’ 5’-CTGTTGTTCAGACTCTCTCCCT-3’

IL-10 5’-GCTCTTACTGACTGGCATGAG-3’ 5’-CGCAGCTCTAGGAGCATGTG-3’

SIRT3 5’-ATCCCGGACTTCAGATCCCC-3’ 5’-CAACATGAAAAAGGGCTTGGG-3’

HIF-1α 5’-ACCTTCATCGGAAACTCCAAAG-3’ 5’-ACTGTTAGGCTCAGGTGAACT-3’

MCP-1 5’-TTAAAAACCTGGATCGGAACCAA-3’ 5’-GCATTAGCTTCAGATTTACGGGT-3’

iNOS 5’-GTTCTCAGCCCAACAATACAAGA-3’ 5’-GTGGACGGGTCGATGTCAC-3’

Arg-1 5’-CTCCAAGCCAAAGTCCTTAGAG-3’ 5’-AGGAGCTGTCATTAGGGACATC-3’

CD206 5’-CTCTGTTCAGCTATTGGACGC-3’ 5’-CGGAATTTCTGGGATTCAGCTTC-3’

β-actin 5’-GGCTGTATTCCCCTCCATCG-3’ 5’-CCAGTTGGTAACAATGCCATGT-3’
