# Supplementary material for: TGR5 deficiency aggravates hepatic ischemic/reperfusion injury via inhibiting SIRT3/FOXO3/HIF-1ɑ pathway
Source: Cell Death Discov. 2020 Nov 1;6:116. doi: 10.1038/s41420-020-00347-2 (PMC7604280; doi:10.1038/s41420-020-00347-2)
Supplement: Supplementary file 2 — Supplementary Table 2 [file 41420_2020_347_MOESM2_ESM.docx]

**Supplementary Table 2: Potential acetylation sites of FOXO3 in mice.**

Lysine residue Species Amino acid sequence

K241 Mus musculus IINPDGGkSGKAPRR

K258 Mus musculus VsMDNSNkYTKSRGR

K270 Mus musculus RGRAAKKkAALQAAP

K289 Mus musculus DsPsQLSkWPGsPTS

K568 Mus musculus SSSLGSAkHQQQsPA
